# Organic Chemistry and Medicine

**Published:** 1847-02

**Authors:** 


*Organic Chemistry and Medicine.*—The point, however, to which I wish to direct your special attention, in reference to our anticipations of advancing the science, is organic chemistry. The apparatus for organic combustion, the great invention of Professor Liebig, is already almost daily in use in our laboratory. By its means, as you know, the analysis of matter belonging to the animal and vegetable kingdoms—the product, in some form or other, of the vital force—is effected with greater ease and equal precision with the analysis of a soil or a mineral. The rapidity with which discoveries are being made, and the immense extent of the science, render it almost impossible, except for the professed chemist, to follow and to appreciate its importance. Every now and then, however, some fact is made known, having an immediate application to practical purposes, and indicating how vast a field of inquiry exists in organic nature. Within the last few days, Professor Liebig has announced to us that a residue left in the manufacture of sulphate of quinine, is the pure alkaloid itself, merely obscured by its form. The discovery of quinine by Pellatier, in 1820, revolutionized pharmacy. The active principles of the most nauseous drugs can now be isolated from the accompaniments of woody fibre and other inert matters, and exhibited in a concentrated form. This is a blessing, which only those who have had to swallow bark in substance can fully understand. During the twenty-six years in which sulphate of quinine has been manufactured, a considerable portion, every year, of the pure alkaloid has been laid aside—and, consequently, a considerable amount has accumulated. Professor Liebig's discovery will not only bring this into use, but it will enable the practitioner of medicine to prescribe the pure alkaloid, and to combine it at pleasure with vegetable or other acids, and thus to obviate the objections which are found in practice to lie against the sulphate of quinine, placing at command quinine and a variety of its salts at a price which will not preclude the poorest from its benefit.

A great portion of the vegetable kingdom still requires to be chemically investigated; of the materials which constitute the animal body, every one, without exception, awaits a new analysis and investigation. Not many months since, Professor Redtenbacher, of Prague, found that taurine, a constituent of bile, which acts a most important part in the animal economy, contains sulphur to the extent of 26 per cent. This element has been previously overlooked, in consequence of what must be considered a defect in the method of analysis of combustion; in that practice the oxygen of the analysed body being estimated by the amount of loss, the equivalent of sulphur being precisely double that of oxygen, its presence did not disturb the calculation of the results. Professor Gregory has remarked, that this observation of Redtenbacher will probably turn out, in its consequences, one of the most important yet made in animal chemistry; and it cannot be doubted, that although our views must be changed in many points in consequence of it, our knowledge will be extended and rendered more precise and more capable of direct application to physiology and pathology. Already Professor Liebig has found reason to believe that Mulder's protein and oxides of protein exist only in imagination, as sulphur remains combined with the other elements after fibrin and albumen are subjected to Mulder's processes. To extend this inquiry into all the materials of the animal tissues, requires many skilful hands and much labor.

This branch of the science is occupying the attention of very many students on the Continent, and is undergoing a rapid development. From the masses of figures and calculations, now accumulating, representing analyses, we may anticipate many practical results of vast importance to emerge.

For my own part, I entertain the most sanguine hopes that the diseases most fatal to mankind will be elucidated, and, perhaps, wholly prevented, by pursuing this path of chemical inquiry.—*London Lancet.*

